# Prevalence and associations of dry eye disease and meibomian gland dysfunction in the ural eye and medical study

**DOI:** 10.1038/s41598-022-22580-8

**Published:** 2022-11-07

**Authors:** Mukharram M. Bikbov, Timur R. Gilmanshin, Rinat M. Zainullin, Gyulli M. Kazakbaeva, Ellina M. Iakupova, Albina A. Fakhretdinova, Azaliia M. Tuliakova, Songhomitra Panda-Jonas, Iuliia A. Rusakova, Ildar F. Nuriev, Artur F. Zaynetdinov, Liana A. Miniazeva, Jost B. Jonas

**Affiliations:** 1 Ufa Eye Research Institute, 90 Pushkin Street, Ufa, Russia 450077; 2 Ufa Eye Institute, Ufa, Russia; 3 Privatpraxis Prof Jonas und Dr Panda-Jonas, Heidelberg, Germany; 4 Department of Ophthalmology, Medical Faculty Mannheim, Heidelberg University, Theodor-Kutzerufer 1, 68167 Mannheim, Germany; 5 Institute of Molecular and Clinical Ophthalmology Basel, Basel, Switzerland

**Keywords:** Diseases, Eye diseases, Corneal diseases

## Abstract

To assess the prevalence of dry eye disease (DED) and Meibomian gland dysfunction (MGD) in a population in Russia. The population-based Ural Eye and Medical Study was conducted in an urban and rural region in Bashkortostan/Russia and included 5899 (80.5%) out of 7328 eligible persons, aged 40 + years. DED and MGD were assessed by Schirmer´s test, slit-lamp based examination of the Meibomian glands, and an interview with DED-related questions. The study included 5153 (87.4%) individuals with DED and MGD assessments (mean age: 58.5 ± 10.5 years). The mean Schirmer´s test result was 11.8 ± 6.8 mm and 12.5 ± 7.1 mm for the right/left eye, with a result of ≤ 5 mm measured in 1098 (21.3%; 95% confidence intervals (CI) 20.2, 22.4) of the worse eyes. The mean subjective dry eye symptoms score was 1.37 ± 1.82. MGD grade 1 (telangiectasia at the lid margin), 2, 3, 4 or any grade in the worse eye was diagnosed in 901 (21.1%), 1161 (27.1%), 158 (3.7%), 32 (0.7%), and 2252 (52.6%; 95% CI 51.1, 54.1) eyes respectively. The prevalence of DED diagnosis #1, #2 and #3 (Schirmer´s test ≤ 5 mm, and dry eye score ≥ 1, ≥ 2, and ≥ 3, respectively), #4 (dry eye score ≥ 1, Schirmer test ≤ 5 mm, MGD grade 1 +), and #5 (dry eye score ≥ 1, Schirmer test ≤ 5 mm, MGD grade 2 +) were 598/5142 (11.6%), 426/5153 (8.3%), 273/5142 (5.3%), 335/5142 (6.5%), and 186/5142 (3.6%), respectively. Mean depression score as applied was 5.1 ± 3.8. Higher DED prevalence (definition #4) was associated (multivariable analysis) with female sex (odds ratio (OR) 1.71; 95% CI 1.31, 2.22; *P* < 0.001), higher depression score (OR 1.04; 95% CI 101, 1.07; *P* = 0.009), and higher prevalence of thyroid disease history (OR 1.63; 95% CI 1.19, 2.24; *P* = 0.006). DED and MGD were common in this rural and urban population, and their prevalence was associated with female sex, thyroid disease, and depression.

## Introduction

Dry eye disorder (DED) and Meibomian gland disease (MGD) belong to the most frequent reasons why patients attend ophthalmologists and optometrists^1,2^. Features of DED as a multifactorial disorder are increased tear film instability with a loss of tear film homeostasis, tear hyperosmolarity, decreased tear production, ocular surface inflammation, and neurosensory abnormalities ^1,3,4^. MGD is characterized by terminal duct obstruction and abnormalities in the glandular secretion and often accompanies or causes DED^5–8^. In previous investigations, the DED prevalence varied in dependence of the diagnostic criteria applied. It ranged between 9 and 30%, when symptoms combined with signs were taken into account, and it ranged between 7 and 52%, when only symptoms were considered^2,9–11^. The MGD prevalence, as estimated in a recent meta-analysis of population-based and clinical studies, was 36% (95% confidence interval (CI): 24, 50), and it was higher in men than in women (odds ratio (OR) 1.24; 95% CI 1.01, 1.52). It was lowest in Africans (21.2%) and Caucasians (29.5%) and highest in Hispanics (67.5%) and Arabs (71.0%)^12–15^. Most of these previous studies consisted of relatively small study samples, and the studies usually examined only few medical disorders in addition to, and in association with, DED and MGD. In particular, none of the previous studies were conducted in Eastern Europe or Russia. In a recent review, Onufriichuk and Kuroyedov from St. Petersburg and Moscow stated that the prevalence of DED is understudied in Russia, with the available investigations performed in Russia varying in quantity and quality, thus preventing conclusions to be mase for the general population in Russia^16–22^. We therefore carried out the present study to examine the prevalence and associations of DED and MGD in a population from Russia. The results of the study should give information on the prevalence of DED in Russia as a world region, and they may show up associations between the prevalence and severity of DED and other ocular and systemic disorders what may be of help for clinicians in their daily taking care of patients with DED and other ocular diseases.

## Methods

### Study participants and inclusion criteria

The individuals included into the present study were the participants of the Ural Eye and Medical Study (UEMS)^23,24^. The UEMS is a population-based investigation which was performed in the Russian republic of Bashkortostan at the southwestern end of the Ural Mountains in the study period from 2015 to 2017^23,24^. Study regions were Ufa as capital of Bashkortostan in a distance of about 1400 km East of Moscow and a rural region in the Karmaskalinsky District in a distance of 65 km from Ufa. With a population of 4 million people, the republic of Bashkortostan located between the Volga River and the Ural Mountains is the most populous republic in Russia. Inclusion criteria for the study were living in the study regions and an age of 40 years or older. The cut-off value of an age of 40 years as inclusion criterion was chosen, since the study was designed to address the prevalence of general and ophthalmological disorders and diseases, the frequencies of most of which increase with older age. Including individuals younger than 40 years would have increased the number of healthy individuals, and would have relatively decreased the percentage of participants affected by disorders. Another reason was the comparability of the results of the present study with those of previous population-based investigations which generally chose an age of 40 years as inclusion criterion for their study populations. The Ethics Committee of the Academic Council of the Ufa Eye Research Institute approved the study design and confirmed that the study adhered to the Declaration of Helsinki (ethics committee approval, dated 25th of August 2015, number #2), and all participants gave an informed written consent. As described in detail recently, out of a total group of 7328 eligible individuals, 5899 (80.5%) individuals (3319 [56.3%] women) with a mean age of 59.0 ± 10.7 years (range: 40–94 years) participated in the study with older age. Includin23,24 g. The study population did not differ significantly in the gender and age distribution from the Russian population as explored in the census carried out in 2010^25^.

### Examinations

As also described in detail previously, all study participants underwent a detailed interview which was conducted by trained social workers and consisted of more than 250 standardized questions on the socio-economic background and lifestyle parameters, depression and anxiety, and known diagnosis and therapy of major diseases^23,24,26–28^. The series of examinations included anthropometry, blood pressure measurement, handgrip dynamometry, spirometry, biochemical analysis of blood samples taken under fasting conditions, and ophthalmologic examinations. The latter consisted of automated refractometry and measurement best-corrected visual acuity, slit lamp-based biomicroscopy of the anterior and posterior ocular segment including the assessment of pseudoexfoliation of the lens in medical mydriasis, digital photography of the cornea, lens, optic nerve head and macula, and spectral-domain optical coherence tomography of the macula and optic nerve head. Besides the conventional fundus photographs, we took red-free fundus photographs using a green filter. Macular pigment optical density was estimated by reflectometry (VISUCAM 500 fundus camera; Carl Zeiss Co, Oberkochen, Germany). As described previously, we differentiated nuclear lens opacities into 6 grades using the classifying scheme for cataract of the Age-Related Eye Disease Study. We defined the presence of nuclear cataract as a nuclear cataract grade of 3 + . Cortical lens opacities were assessed using the photographs taken by retro-illumination. Using the OCT scans, we determined the peripapillary retinal nerve fiber layer thickness, the width and shape of the neuroretinal rim and the depth of the optic cup, and the thickness of the retina as a whole and stratified into various retinal layers in the foveola and the perifoveal region. The degree of fundus tessellation was examined on the fundus photographs centered on the macula and centered on the optic nerve head. Glaucoma was defined by morphological criteria as described by Foster and colleagues^29^. The Guidelines for Accurate and Transparent Health Estimates Reporting (GATHER statement guidelines) for collecting the data were applied^30^. As recommended by the Beckman Initiative for Macular Research Classification Committee, we defined AMD using the fundus photographs^31^. We defined arterial hypertension according to the criteria published by the American Heart Association, and criteria for the diagnosis of diabetes mellitus were a fasting glucose concentration of ≥ 7.0 mmol/L or a self-reported history of physician diagnosis of diabetes mellitus or a history of drug treatment for diabetes (insulin or oral hypoglycemic agents). Anemia was defined by a hemoglobin concentration of < 140 g/L for men and < 130 g/L for women. Depression was assessed by applying the Center for Epidemiologic Studies Depression Scale (CES-D) Scoresheet. The estimated glomerular filtration rate (eGFR) was calculated using the chronic kidney disease (CKD) Epidemiology Collaboration (CKD-EPI) equation.

### Definition of dry eye disease and Meibomian gland disease

As also described in detail previously, the Meibomian glands were assessed by examining the gland orifices and their secretion upon slit-based examination of the anterior segment^32^. We differentiated between “normal” (grade 0), “no obstruction of the Meibomian gland orifices but telangiectasias” (grade 1), “plugged Meibomian gland orifices with translucent serous secretion when the lid margin was compressed” (grade 2), “plugged Meibomian gland orifices with viscous or waxy white secretion when the lid margin was compressed” (grade 3), and “plugged Meibomian gland orifices and no secretion when the lid margin was compressed” (grade 4). Both eyes of each study participant were examined, and the data of the worse eye were used for the statistical analysis. The prevalence and degree of a DED were assessed by specific questions in the questionnaire and by additional physical examinations. The questions were: (1) Do your eyes feel dry; (2) Do you ever feel a gritty or sandy sensation in your eye; (3) Do your eyes ever have a burning sensation; (4) Are your eyes ever red; (5) Do you notice much crusting on your lashes; and (6) Do your eyes ever get stuck in the morning. All questions were answered using a scale of grade 0 for “never”, grade 1 for “rarely or sometimes”, and grade 2 for “frequently or always”. A quantitative grading score of the subjective dry eye symptoms was obtained by summarizing the grades of the answers to the six questions (0–12). As described by Wolffsohn and colleagues, we performed a Schirmer test without the use of a topical anesthetic drug^2^. We folded a Schirmer paper strip (5 × 35 mm) at the notch and hooked the folded end over the temporal one-third of the lower lid margin. The participants were asked to close their eyes, and after five minutes, the strip was removed and the length of wetting from the notch was measured. For the diagnosis of a DED, several definitions were used. Definition #1 was made with a dry eye symptom score of ≥ 1 and a Schirmer´s test ≤ 5 mm. In definition #2, the dry eye symptoms score was ≥ 2 and Schirmer´s test ≤ 5 mm, and in definition #3, the dry eye symptoms score was ≥ 3 with a Schirmer´s test of ≤ 5 mm. In definition #4, the dry eye symptoms score was ≥ 1, with a Schirmer test ≤ 5 mm and a MGD grade of 1 (telangiectasia at the lid margin) or higher. In definition #5, the dry eye symptoms score was ≥ 1, Schirmer test ≤ 5 mm and the MGD grade was 2 (plugged Meibomian gland orifices with translucent serous secretion when the lid margin was compressed) or higher^32,33^.

### Statistical analysis

The statistical analysis was conducted using a commercially available statistical software package (SPSS for Windows, version 27.0, SPSS, Chicago, IL). Inclusion criteria for the present study were the availability of measurements of the Schirmer test, examination of the Meibomian glands and assessment of the dry eye symptoms. The data of the worse eye per study participant is taken for the statistical analysis. We first calculated the mean values (presented as mean and 95% confidence intervals (CI) or as mean ± standard error) of the main outcome parameters. We then conducted a univariate logistic regression analysis of the associations between the presence of a DED or MGD and other ocular and systemic parameters. A subsequent binary multivariable regression analysis included the presence of DED and MGD as the dependent parameter and as independent parameters all those variables that were associated (*P* ≤ 0.10) with the DED and MGD presence in the univariable analyses. We chose a cut-off value of ≤ 0.10 to reduce the risk of missing a parameter the association of which might have become statistically significant in the eventual multivariable analysis. In a step-by-step manner, we dropped those variables out of the list of independent parameters that were no longer significantly associated with the DED and MGD prevalence. We determined the odds ratios (ORs) and their 95% CIs. All *P*-values were two-sided and considered statistically significant when the values were less than 0.05.

## Results

Out of the 5899 participants of the Ural Eye and Medical Study, the present investigation included 5153 (87.4%) individuals (2803 [54.4%] women) with assessments of dry eye symptoms including a bilateral Schirmer´s test. The mean age was 58.5 ± 10.5 years (median: 58 years; range 40–94 years), and the mean axial length was 23.3 ± 1.1 mm (median: 23.24 mm; range: 19.78–32.87 mm). The group of study participants as compared with the group of individuals without dry eye assessments was younger (58.5 ± 10.5 years versus 62.2 ± 11.5 years; *P* < 0.001), showed a lower (*P* < 0.001) proportion of women versus men (2803/2350 versus 516/230), and had a longer axial length (23.3 ± 1.1 mm versus 23.2 ± 1.2 mm; *P* = 0.006). Stratifying the study population by age and gender revealed an age-related increase in the percentage of women: age group of 40 to < 45 years (n = 436; 236 (54.1%) women), 45 to < 50 years (n = 685; 349 (50.9%) women), 50 to < 55 years (n = 856; 453 (52.9%) women), 55 to < 60 years (n = 943; 481 (51.0%) women), 60 to < 65 years (n = 794; 424 (53.4%) women), 65 to < 70 years (n = 666; 403 (60.5%) women), 70 to < 75 years (n = 278; 167 (60.1%) women), 75 to < 80 years (n = 337; 194 (57.6%) women) and 80 + years (n = 158; 96 (60.8%) women).

**Figure 1:**
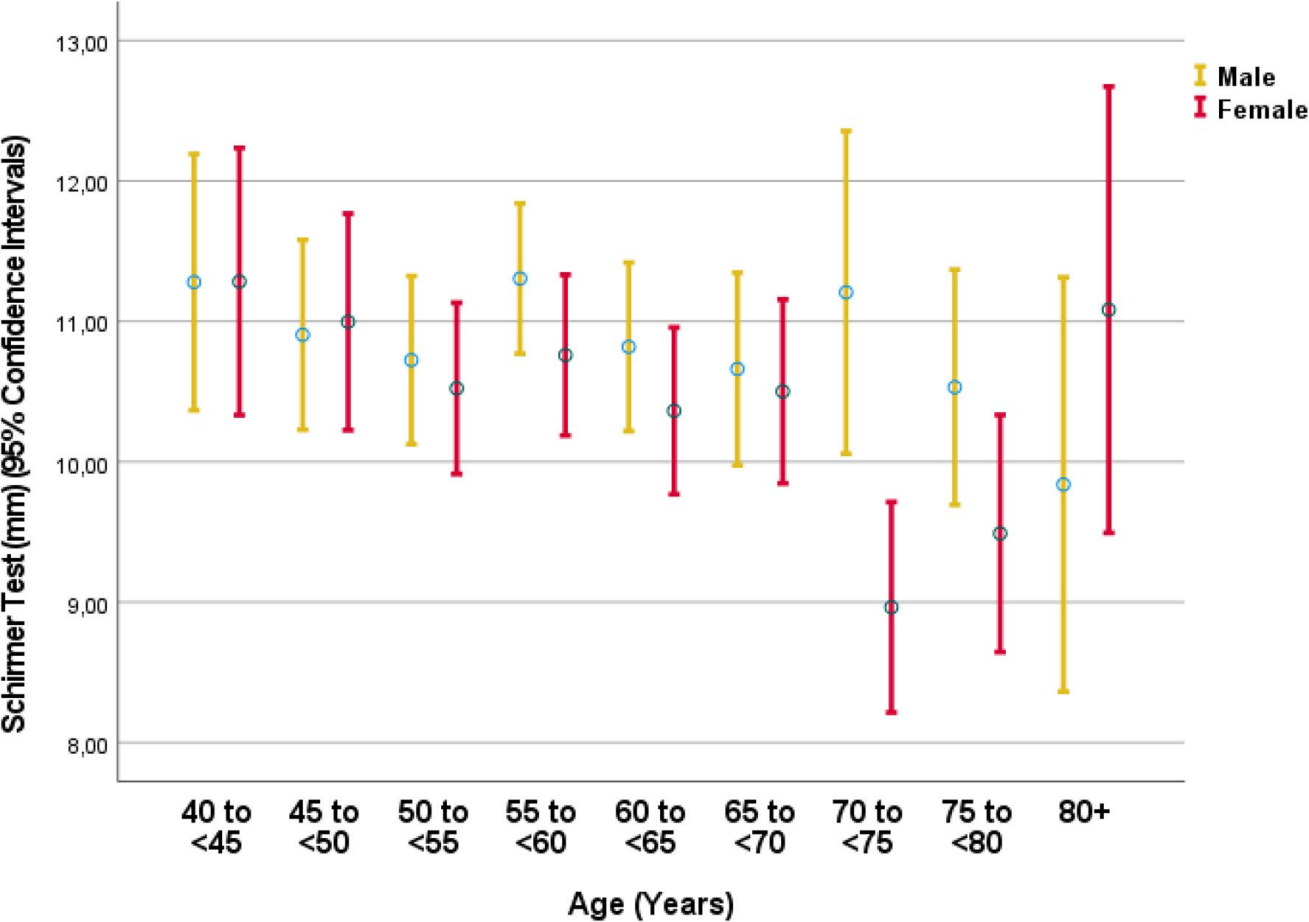
Graph showing the distribution of the Schirmer´s test result stratified by age and sex in the Ural Eye and Medical Study.

**Figure 2:**
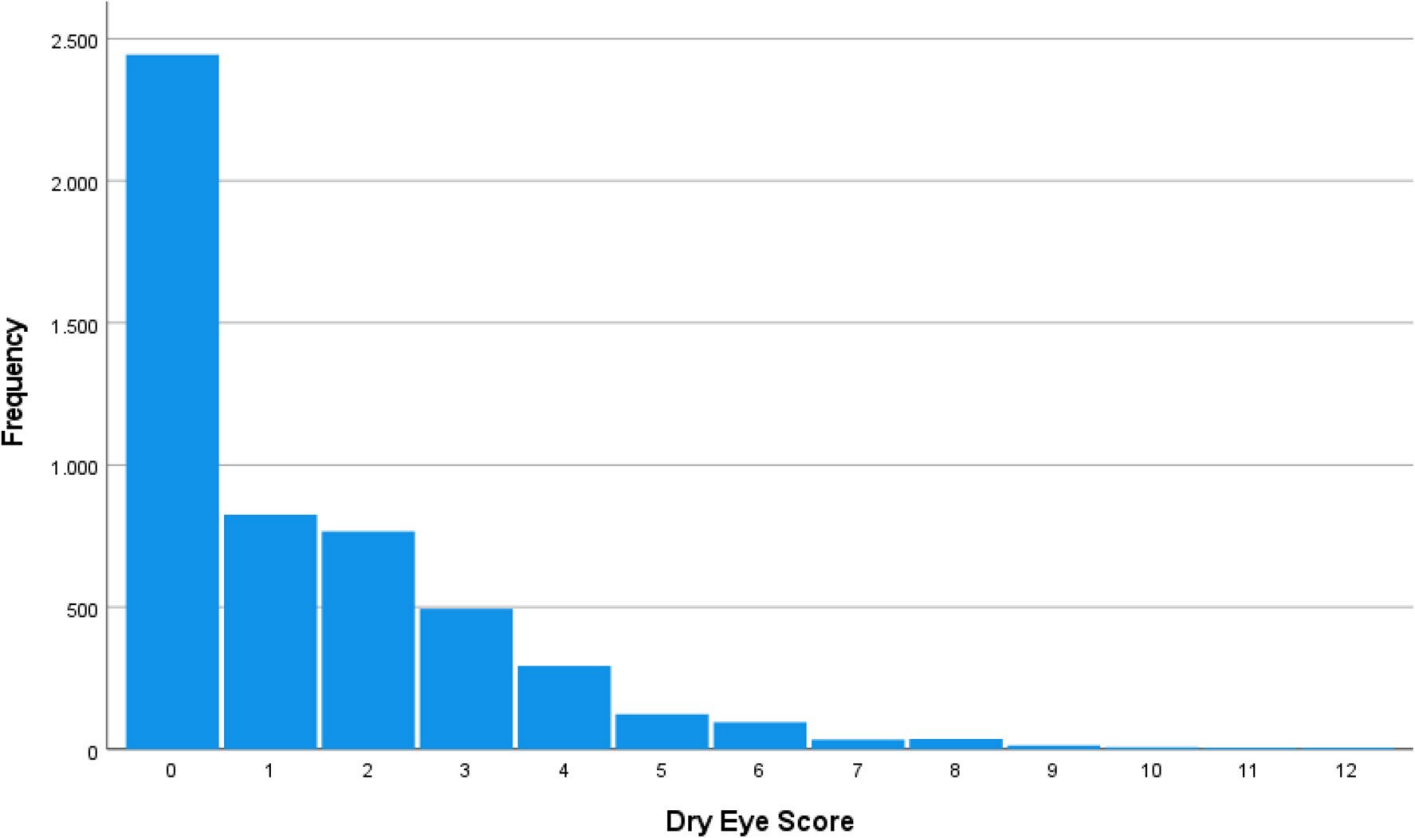
Histogram showing the distribution of the total score of the dry eye symptoms in the Ural Eye and Medical Study The prevalence and degree of a DED were assessed by specific questions in the questionnaire and by additional physical examinations. The questions were: 1) Do our eyes feel dry; 2) Do you ever feel a gritty or sandy sensation in your eye; 3) Do your eyes ever have a burning sensation; 4) Are your eyes ever red; 5) Do you notice much crusting on your lashes; and 6) Do your eyes ever get stuck in the morning. All questions were answered using a scale of grade 0 for “never”, grade 1 for “rarely or sometimes”, and grade 2 for “frequently or always”. A quantitative grading score of the subjective dry eye symptoms was obtained by summarizing the grades of the answers to the six questions (0–12).

**Figure 3:**
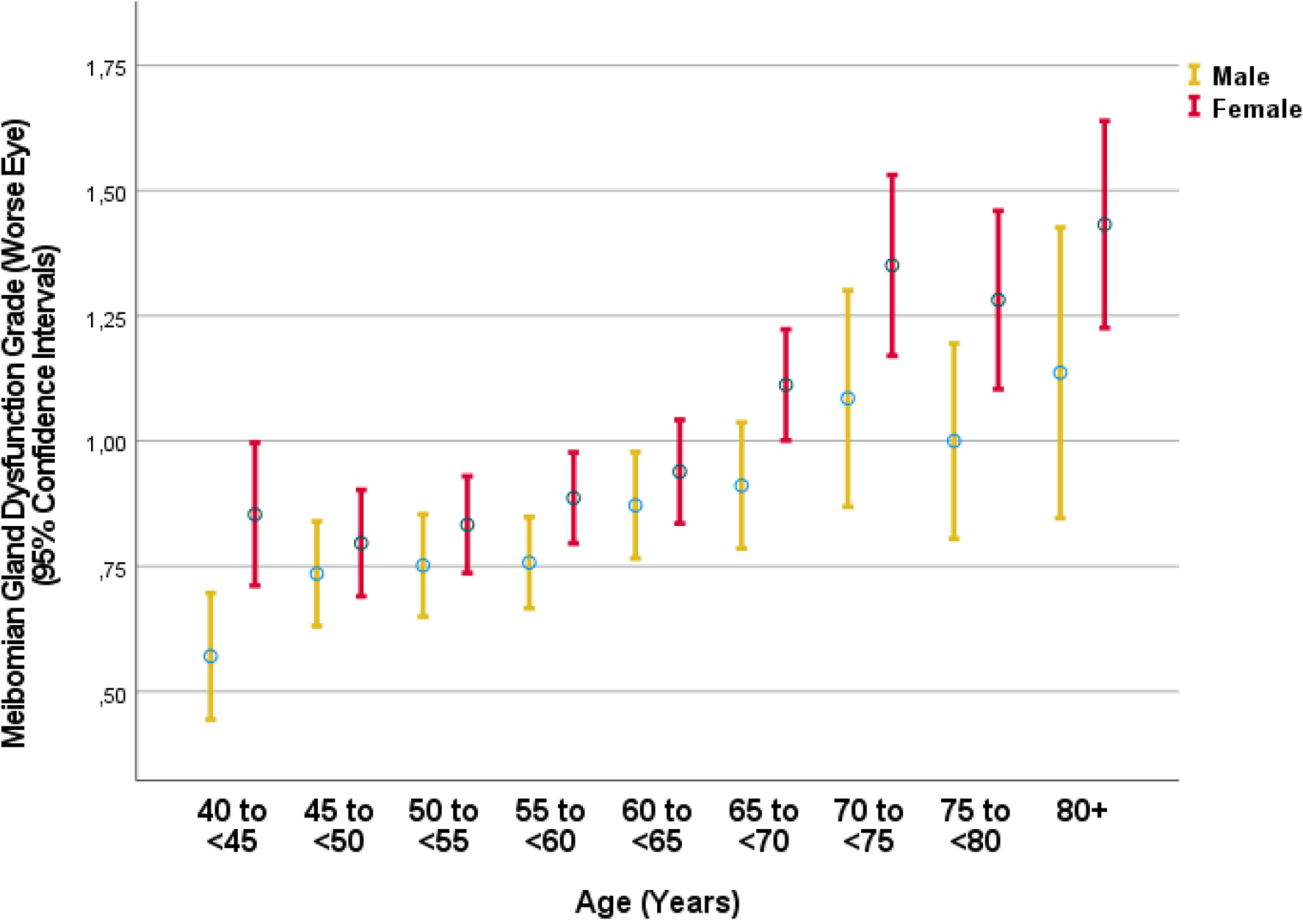
Graph showing the distribution of Meibomian gland dysfunction stratified by age and sex in the Ural Eye and Medical Study.

The mean result of the Schirmer´s test was 11.8 ± 6.8 mm (median 10; range: 0–35 mm) in the right eye and 12.5 ± 7.1 mm (median 12; range: 0–35 mm) in the left eye (Fig. 1). A Schirmer´s test of ≤ 5 mm was measured in 892 (17.3%; 95% CI 16.3, 18.3) right eyes, 786 (15.3%; 95% CI 14.3, 16.2) left eyes, and in 1098 (21.3%; 95% CI 20.2, 22.4) of the worse eyes (defined as the eye with the smaller Schirmer test result). The mean score of the subjective dry eye symptoms was 1.37 ± 1.82 (median: 1; range: 0–12) (Fig. 2). An MGD grade 1, 2, 3 and 4 in the worse eye was diagnosed in 901 (21.1%), 1161 (27.1%), 158 (3.7%), and 32 (0.7%) eyes, respectively (Table 1) (Fig. 3). The prevalence of MGD of any degree was 52.6% (95% CI 51.1, 54.1). The prevalence of DED diagnosis 1, 2, 3, 4, and 5 were 598/5142 (11.6%), 426/5153 (8.3%), 273/5142 (5.3%), 335/5142 (6.5%), and 186/5142 (3.6%), respectively (Table 2).

**Table 1:** Prevalence of dry eye symptoms.

	Never (grade 0)	Rarely or sometimes (grade 1)	Frequently or always (grade 2)
Do your eyes feel dry?	4162 (80.8%)	827 (16.0%)	164 (3.2%)
Do you ever feel a gritty or sandy sensation in your eye? (missing: 0)	3688 (71.6%)	1268 (24.6%)	195 3.8%)
Do your eyes ever have a burning sensation? (missing: 0)	3994 (77.5%)	1034 20.1%)	125 2.4%)
Are your eyes ever red?	3400 (66.0%)	1509 (29.3%)	238 (4.6%)
Do you notice much crusting on your lashes? (missing: 0)	4583 (88.9%)	507 (9.8%)	62 1.2%)
Do your eyes ever get stuck in the morning? (missing: 0)	4857 (94.3%)	246 (4.8%)	46 0.9%)

**Table 2:** Prevalence of Meibomian gland dysfunction.

	Normal	No obstruction, but telangiectasias (grade 1)	Plugged with translucent serous secretion when the lid margin was compressed (grade 2)	Plugged with viscous or waxy white secretion when the lid margin was compressed (grade 3)	Plugged with no secretion when the lid margin was compressed (grade 4)	Meibomian gland dysfunction, any degree
Right eye (missing: 863)	2029 (47.3%)	906 (21.1%)	1165 (27.2%)	158 (3.7%)	32 (0.7%)	2261 (52.7%; 95% CI 51.2, 54.2)
Left Eye (missing 32###5)	2030 47.4%)	905 (21.1%)	1161 (27.1%)	157 (3.0%)	31 (0.6%)	2254 (52.6%; 95% CI 51.1, 54.1)
Worse eye (missing 873)	2028 (47.4%)	901 (21.1%)	1161 (27.1%)	158 (3.7%)	32 (0.7%)	2252 (52.6%; 95% CI 51.1, 54.1)

**Figure 4:**
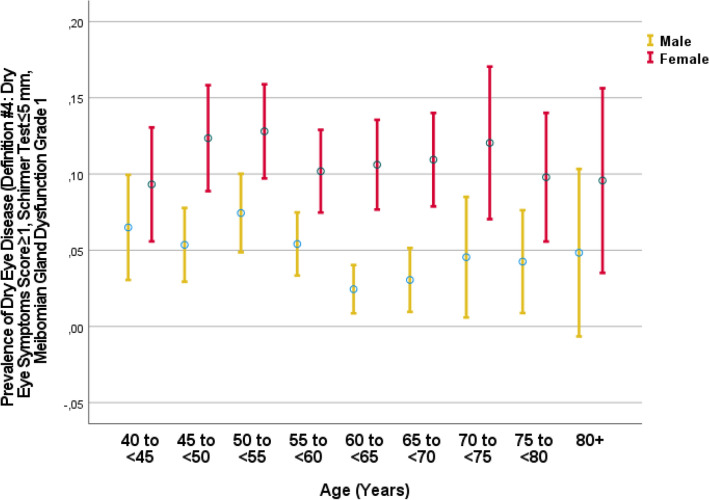
Graph showing the distribution of the prevalence of dry eye disease (definition #2: dry eye symptoms score was ≥ 2 and Schirmer´s test of < 5 mm) stratified by age in the Ural Eye and Medical Study.

**Figure 5:**
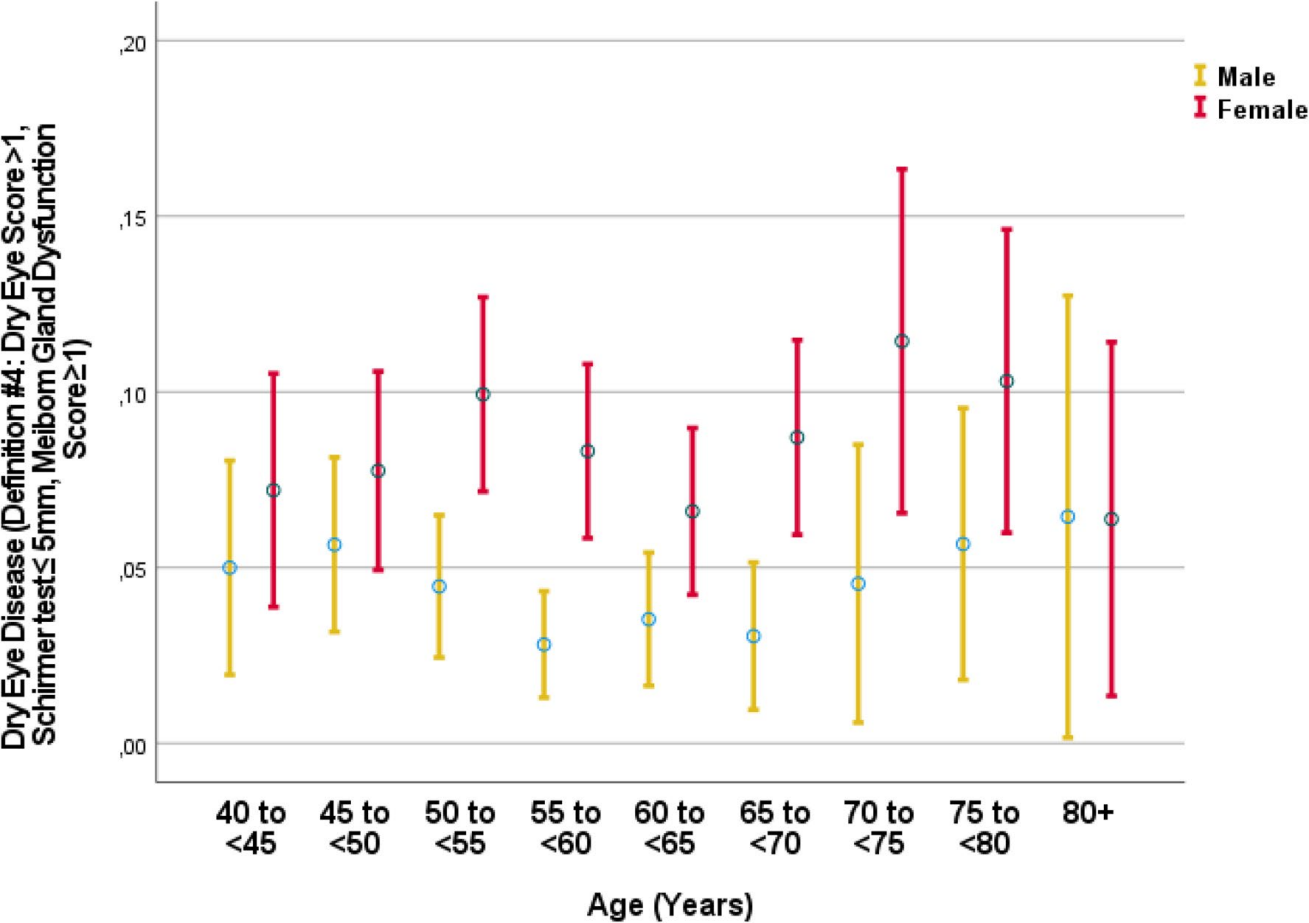
Graph showing the distribution of the prevalence of dry eye disease (definition #4: dry eye symptoms score was ≥ 1, Schirmer test ≤ 5 mm, and a Meibomian gland dysfunction grade of 1 (telangiectasia at the lid margin) or higher) stratified by age in the Ural Very Old Study.

In univariable analysis, a higher prevalence of DED definitions #2 and #4 was associated with the systemic parameters of female sex, region of habitation, higher body height and weight, higher hip circumference, lower waist-hip circumference ratio, higher socioeconomic score, lower prevalence of any alcohol consumption and current smoking and lower smoking package years, lower number of days per week with fruit intake, higher prevalence of a history of cardiovascular disorders and angina pectoris, arthritis, neck pain, headache, thoracic spine pain, diarrhea, osteoarthritis, thyroid disease and previous falls, lower serum concentration of bilirubin and hemoglobin, higher serum concentration of high-density lipoproteins, cholesterol and urea, higher erythrocyte sedimentation rate, lower count of erythrocytes and leukocytes, lower percentage of rod-core granulocytes and segment nuclear granulocytes and higher percentage of monocytes, higher prevalence of anemia and chronic obstructive pulmonary disease, higher depressions core, anxiety score, and lower dynamometric hand grip force (Table 3) (Fig. 4, 5). A higher prevalence of DED definitions #2 and 4 was associated with the ocular parameters of shorter axial length, higher corneal refractive power, and higher corneal volume, lower intraocular pressure, higher prevalence of nuclear cataract, and any cataract, lower degree of peripapillary fundus tessellation, lower retinal thickness 300 µm nasal to the fovea, higher degree of lens pseudoexfoliation, and lower diabetic retinopathy grading (Table 4).

**Table 3:** Associations (binary univariable analysis) between the prevalence of dry eye disease using definition #2 (dry eye symptoms score was ≥ 2 and Schirmer´s test of < 5 mm) or definition #4 (dry eye symptoms score was ≥ 1, Schirmer test ≤ 5 mm, and a Meibomian gland dysfunction grade of 1 (telangiectasia at the lid margin) or higher) with systemic parameters in the Ural Eye and Medical Study.

Parameter	Measurement unit	Definition #2		Definition #4	
		Standardized regression coefficient or odds ratios (95% confidence intervals)	*P*-value	Standardized regression coefficient or odds ratios (95% confidence intervals)	*P*-value
Age	1-year intervals	− 0.01 (0.99, 1.00)	0.28	0.01 (0.99, 1.02)	0.37
Gender	Men/Women	2.36 (1.90, 2.95)	< 0.001	2.12 (1.67, 2.70)	< 0.001
Region of habitation	Rural/Urban	1.19 (0.97, 1.46)	0.09	1.19 (0.95, 1.49)	0.14
Ethnicity	Any other ethnicity/Russian	0.97 (0.76, 1.24)	0.90	0.98 (0.75, 1.28)	0.95
Body height	1 cm	0.97 (0.95, 0.98)	< 0.001	0.96 (0.95, 1.00)	< 0.001
Body weight	kg	0.99 (0.98, 0.99)	0.002	0.99 (0.98, 1.00)	0.02
Body mass index	kg/m^2^	1.00 (0.98, 1.02)	0.70	1.01 (0.99, 1.03)	0.48
Waist circumference	cm	1.00 (0.99, 1.00)	0.21	1.00 (0.99, 1.01)	0.64
Hip circumference	cm	1.01 (1.00, 1.02)	0.04	1.01 (1.00, 1.02)	0.08
Waist/hip circumference ratio	Ratio	0.08 (0.02, 0.24)	< 0.001	0.14 (0.04, 0.52)	0.003
Socio-economic Score	Score	1.07 (1.00, 1.15)	0.04	1.05 (0.97, 1.13)	0.25
Level of education*	1–8	1.05 (0.98, 1.12)	0.20	1.01 (0.94, 1.10)	0.74
Physical activity Score	Score	1.00 (0.99, 1.01)	0.79	0.99 (0.98, 1.01)	0.37
Smoking, currently	Yes/No	0.65 (0.46, 0.91)	0.01	0.4 (0.51, 1.06)	0.11
Smoking, package years	Number	0.99 (0.98, 1.00)	0.07	1.00 (0.99, 1.01)	0.32
Alcohol consumption, any	Yes/No	0.68 (0.52, 0.88)	0.003	0.66 (0.49, 0.88)	0.005
In a week how many days do you eat fruits?	Number of days	1.08 (1.02, 1.14)	0.006	1.05 (0.99, 1.11)	0.09
In a week how many days do you eat vegetables?	Number of days	1.06 (0.98, 1.14)	0.15	1.07 (0.98, 1.16)	0.15
History of cardiovascular disorders including stroke	Yes/No	1.25 (1.01, 1.56)	0.04	1.25 (0.99, 1.59)	0.07
History of angina pectoris	Yes/No	1.86 (1.39, 2.49)	< 0.001	1.64 (1.18, 2.29)	0.005
History of asthma	Yes/No	0.51 (0.22, 1.16)	0.11	0.90 (0.44, 1.86)	1.00
History of arthritis	Yes/No	1.33 (1.07, 1.65)	0.01	1.13 (0.88, 1.44)	0.34
History of previous bone fractures	Yes/No	0.94 (0.76, 1.17)	0.58	1.01 (0.80, 1.29)	0.95
History of low back pain	Yes/No	1.06 (0.87, 1.30)	0.58	1.08 (0.87, 1.35)	0.50
History of thoracic spine pain	Yes/No	1.37 (1.10, 1.71)	0.006	1.02 (1.00, 1.77)	0.01
History of neck pain	Yes/No	1.37 (1.11, 1.69)	0.004	1.19 (0.94, 1.50)	0.17
History of headache	Yes/No	1.41 (1.16, 1.72)	0.001	1.13 (0.91, 1.41)	0.28
History of cancer	Yes/No	0.74 (0.37, 1.46)	0.44	0.73 (0.34, 1.58)	0.60
History of dementia	Yes/No	0.32 (0.04, 2.30)	0.36	0.41 (0.06, 2.99)	0.73
History of diarrhea	Yes/No	1.51 (0.45, 5.08)	0.46	2.75 (09.4, 8.07)	0.08
History of iron-deficiency anemia	Yes/No	1.17 (0.78, 1.76)	0.44	0.62 (0.34, 1.11)	0.11
History of low blood pressure and hospital admittance	Yes/No	1.34 (0.82, 2.22)	0.25	0.81 (0.41, 1.60)	0.64
History of osteoarthritis	Yes/No	1.33 (1.05, 1.69)	0.02	1.29 (0.98, 1.68)	0.07
History of skin disease	Yes/No	1.20 (0.79, 1.82)	0.43	1.16 (0.73, 1.86)	0.53
History of thyroid disease	Yes/No	2.08 (1.58, 2.73)	< 0.001	2.20 (1.64, 2.96)	< 0.001
History of falls	Yes/No	1.27 (1.00, 1.61)	0.056	1.20 (0.92, 1.56)	0.20
History of unconsciousness	Yes/No	1.09 (0.77, 1.55)	0.64	1.04 (0.70, 1.56)	0.84
Age of the last menstrual bleeding	Years	1.01 (0.98, 1.04)	0.54	1.02 (0.99, 1.05)	0.30
Age of last regular menstrual bleeding	Years	1.00 (0.98, 1.03)	0.75	1.02 (0.99, 1.05)	0.33
History of menopause	Yes/No	1.00 (0.74, 1.35)	1.00	1.21 (0.84, 1.73)	0.34
History of diabetes mellitus	Yes/No	1.13 (0.80, 1.56)	0.46	1.01 (0.68, 1.51)	0.92
**Serum concentration of:**					
Alanine aminotransferase	IU/L	1.00 (0.99, 1.00)	0.30	1.00 (0.99, 1.01)	0.36
Aspartate aminotransferase	IU/L	0.99 (0.98, 1.00)	0.12	0.99 (0.98, 1.00)	0.20
Aspartate aminotransferase-to- Alanine aminotransferase ratio		0.83 (0.57, 1.21)	0.33	0.81 (0.53, 1.24)	0.34
Bilirubin, total	µmol/L	0.99 (0.98, 1.00)	0.08	0.98 (0.97, 0.99)	0.004
High-density lipoproteins	mmol/L	1.13 (1.02, 1.26)	0.02	1.22 (1.09, 1.37)	0.001
Low-density lipoproteins	mmol/L	1.04 (0.96, 1.12)	0.37	0.99 (0.90, 1.08)	0.99
Cholesterol	mmol/L	1.05 (1.01, 1.11)	0.03	1.05 (1.00, 1.11)	0.049
Triglycerides	mmol/L	1.05 (0.93, 1.19)	0.45	1.08 (0.95, 1.24)	0.24
Rheumatoid factor	IU/mL	1.03 (0.93, 1.15)	0.56	1.08 (0.98, 1.20)	0.13
Erythrocyte sedimentation rate	Mm/min	1.01 (1003, 1.02)	0.01	1.02 (1.01, 1.03)	< 0.001
Glucose	mmol/L	1.02 (0.97, 1.08)	0.44	0.99 (0.93, 1.06)	0.83
Urea	mmol/L	1.11 (1.05, 1.18)	< 0.001	1.20 (1.13, 1.27)	< 0.001
Creatinine	µmol/L	1.00 (0.99, 1.002)	0.21	1.00 (1.00, 1.00)	0.99
Hemoglobin	g/L	0.99 (0.98, 1.00)	0.005	0.99 (0.98, 0.99)	< 0.001
Erythrocyte count	10^6^ cells/µL	0.68 (0.53, 0.88)	0.004	0.54 (0.41, 0.72)	< 0.001
Leukocyte count	10^9^ cells/L	0.93 (0.86, 1.00)	0.046	0.91 (0.84, 0.99)	0.03
Rod-core granulocytes	% of leukocytes	1.07 (1.00 1.15)	0.044	1.10 (1.02, 1.19)	0.01
Segment nuclear granulocyte	% of leukocytes	0.99 (0.98, 1.01)	0.23	0.98 (0.97, 1.00)	0.02
Eosinophil granulocytes	% of leukocytes	0.99 (0.89, 1.09)	0.77	1.05 (0.96, 1.14)	0.32
Lymphocytes	% of leukocytes	0.99 (0.98, 1.01)	0.37	1.00 (0.98, 1.02)	0.80
Monocytes	% of leukocytes	1.05 (1.01, 1.09)	0.02	1.10 (1.05, 1.15)	< 0.001
Prevalence of diabetes mellitus	Yes/No	1.06 (0.78, 1.45)	0.69	0.94 (0.66, 1.35)	0.86
Estimated glomerular filtration rate	30 mL/min/1.73m^2^	1.00 (0.99, 1.00)	0.59	1.00 (0.99, 1.00)	0.23
Stage of chronic kidney disease	0–5	1.00 (0.99, 1.02)	0.62	1.01 (0.99, 1.02)	0.41
Anemia	Yes/No	1.16 (0.92, 1.45)	0.21	1.34 (1.05, 1.72)	0.02
Blood pressure, systolic (SBP)	mm Hg	1.00 (1.00, 1.01)	0.87	1.00 (0.996, 1.01)	0.65
Blood pressure, diastolic (DBP)	mm Hg	1.00 (0.99, 1.01)	0.36	1.00 (0.99, .1.01)	0.57
Blood pressure, mean	mm Hg	1.00 (0.99, 1.01)	0.67	1.00 (0.99, 1.01)	0.94
Arterial hypertension	Yes/No	0.88 (0.65, 1.20)	0.42	1.06 (0.74, 1.51)	0.86
Arterial hypertension, stage	0–4	0.97 (0.88, 1.06)	0.48	1.02 (0.92, 1.14)	0.71
Prevalence of chronic obstructive pulmonary disease	Yes/No	1.58 (1.12, 2.23)	0.01	1.44 (0.97, 2.13)	0.09
Hearing loss	Hearing loss score (0–44)	1.00 (0.99, 1.01)	0.65	1.00 (0.99, 1.01)	0.93
Depression Score	Depression score unit (range −4 to + 15)	1.06 (1.03, 1.09)	< 0.001	1.06 (1.04, 1.09)	< 0.001
State-Trait Anxiety Inventory	State-Trait Anxiety Inventory Score (range −7 to 13)	1.06 (1.03, 1.09)	< 0.001	1.06 (1.03, 1.09)	< 0.001
Manual dynamometry, right hand	dekaNewton	0.97 (0.96, 0.98)	< 0.001	0.97 (0.96, 0.98)	< 0.001
Manual dynamometry, right hand	dekaNewton	0.97 (0.96, 0.98)	< 0.001	0.97 (0.96, 0.98)	< 0.001

*Level of education was graded into 8 grades: illiteracy/passing 5^th^ grade/8th grade/10th grade/11th grade/graduates/specialized secondary education/post graduates.

**Table 4:** Associations (binary univariable analysis) between the prevalence of dry eye disease using definition #2 (dry eye symptoms score was ≥ 2 and Schirmer´s test of < 5 mm) or definition #4 (dry eye symptoms score was ≥ 1, Schirmer test ≤ 5 mm, and a Meibomian gland dysfunction grade of 1 (telangiectasia at the lid margin) or higher) with ocular parameters in the Ural Eye and Medical Study.

Parameter	Measurement unit	Definition #2		Definition #4	
		Standardized regression coefficient or odds ratios (95% confidence intervals)	*P*-value	Standardized regression coefficient or odds ratios (95% confidence intervals)	*P*-value
Visual acuity, besi corrected, betetr eye	logMAR (minial angle of resolution)	0.92 (0.56, 1.52)	0.74	1.22 (0.78, 1.93)	0.39
Refractive error, spherical equivalent	Diopters	0.98 (0.94, 1.03)	0.46	1.01 (0.96, 2.06)	0.73
Refractive error, cylindrical value	Diopters	1.01 (0.89, 1.15)	0.90	1.01 (0.87, 1.17)	0.90
Axial length	mm	0.87 (0.79, 0.96)	0.006	0.90 (0.81, 1.01)	0.07
Corneal refractive power	Diopters	1.11 (1.04, 1.18)	0.001	1.06 (0.99, 1.14)	0.09
Central corneal thickness	µm	1.00 (1.00, 1.00)	0.54	1.00 (0.995, 1.00)	0.30
Corneal volume	mm^3^	1.02 (1.00, 1.05)	0.10	1.00 (0.98, 1.03)	0.81
Anterior chamber depth	mm	0.95 (0.77, 1.17)	0.63	1.07 (0.85, 1.34)	0.58
Anterior chamber volume	µL	1.00 (1.00, 1.001)	0.16	1.00 (1.00, 1.00)	0.49
Anterior chamber angle	Degree	1.00 (0.99, 1.01)	0.99	1.00 (0.98, 1.01)	0.51
Lens thickness	mm	0.90 (0.71, 1.15)	0.41	1.00 (0.76, 1.31)	1.00
Intraocular Pressure	mmHg	0.99 (0.97, 1.02)	0.48	0.97 (0.94, 1.00)	0.04
Nuclear cataract degree	Grade	1.04 (0.94, 1.15)	0.42	1.09 (0.97, 1.22)	0.14
Nuclear cataract, presence	Yes/No	1.14 (0.93, 1.41)	0.22	1.34 (1.06, 1.69)	0.01
Cortical cataract, degree	Percentage	1.00 (0.99, 1.01)	0.60	1.00 (0.99 1.01)	0.58
Cortical cataract, presence	Yes/No	1.05 (0.79, 1.40)	0.77	1.14 (0.83, 1.55)	0.45
Subcapsular cataract, degree	Percentage	1.01 (0.97, 1.05)	0.59	1.01 (0.97, 1.06)	0.61
Subcapsular cataract, presence	Yes/No	1.23 (0.37, 4.09)	0.73	1.03 (0.25, 4.36)	1.00
Any cataract, presence	Yes/No	1.14 (0.93, 1.39)	0.22	1.32 (1.06, 1.65)	0.02
Status after cataract surgery	Yes/No	0.84 (0.48, 1.45)	0.60	1.01 (0.57, 1.79)	1.00
Fundus tessellation, macula region	Grade	0.91 (0.80, 1.03)	0.14	1.01 (0.88, 1.16)	0.88
Fundus tessellation, peripapillary region	Grade	0.90 (0.81, 1.00)	0.06	0.95 (0.85, 1.07)	0.43
Retinal thickness (total), fovea	µm	1.00 (1.00, 1.00)	0.62	1.00 (1.00, 1.00)	0.49
Retinal thickness (total), 300 µm temporal to the fovea	µm	1.00 (1.00, 1.00)	0.35	1.00 (1.00, 1.00)	0.56
Retinal thickness (total), 300 µm nasal to the fovea	µm	1.00 (1.00, 1.00)	0.06	1.00 (1.00, 1.00)	0.07
Retinal nerve fiber layer thickness	µm	1.00 (0.99, 1.01)	0.94	1.00 (0.99, 1.00)	0.52
Pseudoexfoliation of the lens, degree	0–6	1.09 (0.96, 1.23)	0.18	1.13 (1.00, 1.29)	0.06
Pseudoexfoliation of the lens, presence	Yes/No	1.32 (0.89, 1.97)	0.17	1.39 (0.89, 2.14)	0.14
Glaucoma, presence	Yes/No	0.68 (0.41, 1.13)	0.14	0.67 (0.37, 1.20)	0.18
Glaucoma stage (0–5)	0–5	0.75 (0.53, 1.05)	0.10	0.78 (0.55, 1.13)	0.19
Open-angle glaucoma, presence	Yes/No	0.73 (0.44, 1.22)	0.23	0.68 (0.35, 1.32)	0.25
Angle-closure glaucoma, presence	Yes/No	0.51 (0.17, 1.56)	0.24	0.63 (0.24, 1.69)	0.36
Diabetic retinopathy, ETDRS grading		1.00 (0.99, 1.00)	0.02	1.00 (0.99, 1.00)	0.01
Diabetic retinopathy, presence	Yes/No	1.43 (0.76, 2.71)	0.27	1.47 (0.73, 2.94)	0.28
Myopic maculopathy, stage	0–4	0.81 (0.56, 1.16)	0.25	0.87 (0.59, 1.27)	0.46
Myopic maculopathy, presence (stage 2 +)	Yes/No	0.91 (0.36, 2.27)	1.00	0.68 (0.21, 2.18)	0.80
Age-related macular degeneration (AMD), presence	Yes/No	0.79 (0.54, 1.16)	0.28	0.91 (0.61, 1.36)	0.77
AMD, early stage	Yes/No	0.92 (0.59, 1.43)	0.83	0.84 (0.51, 1.39)	0.55
AMD, intermediate stage	Yes/No	0.63 (0.29, 1.36)	0.33	1.19 (0.62, 2.30)	0.59

In the multivariable analysis with the prevalence of DED definition #4 as the dependent variable, we dropped, due to lack of statistical significance, the parameters of current smoking (*P* = 0.82), serum concentration of cholesterol (*P* = 0.90), waist-hip circumference ratio (*P* = 0.91), history of osteoarthritis (*P* = 0.77) and falls (*P* = 0.82), neck pain (*P* = 0.70), segment nuclear granulocytes (*P* = 0.85), prevalence of anemia (*P* = 0.77), axial length (*P* = 0.91), prevalence of nuclear cataract (*P* = 0.92), corneal refractive power (*P* = 0.54), peripapillary fundus tessellation (*P* = 0.48), history of cardiovascular diseases (*P* = 0.81) and diarrhea (*P* = 0.47), prevalence of chronic obstructive pulmonary disease (*P* = 0.72), erythrocyte count (*P* = 0.48), stage of diabetic retinopathy (*P* = 0.63), anxiety score (*P* = 0.73), history of arthritis (*P* = 0.39), hemoglobin concentration (*P* = 0.32), erythrocyte sedimentation rate (*P* = 0.38), corneal volume (*P* = 0.26), sex (*P* = 0.95), grade of lens pseudoexfoliation (*P* = 0.30), retinal thickness 300 µm nasal to the fovea (*P* = 0.21), hand grip force (*P* = 0.29), region of habitation (*P* = 0.26), leukocyte count (*P* = 0.26), body weight (*P* = 0.16), history of headache (*P* = 0.16), hip circumference (*P* = 0.14), thoracic spine pain (*P* = 0.15), any alcohol consumption (*P* = 0.10), smoking package years (*P* = 0.11), socioeconomic score (*P* = 0.11), prevalence of any cataract (*P* = 0.10), high-density lipoproteins (*P* = 0.09), rod core granulocytes (*P* = 0.08). In the final model, a higher prevalence of DED (definition #4) was associated with female sex, higher depression score, higher number of days per week with fruit intake, higher prevalence of a history of thyroid disease, higher serum concentration of urea and lower bilirubin concentration, higher monocyte percentage, and lower intraocular pressure (Table 5). Adding the parameter of age (*P* = 0.49), region of habitation (*P* = 0.11), body mass index (*P* = 0.70), waist-hip circumference ratio (*P* = 0.37), physical activity score (*P* = 0.67), prevalence of current smoking (*P* = 0.30), level of education (*P* = 0.35), prevalence of diabetes mellitus (*P* = 0.42), history of angina pectoris (*P* = 0.06) and axial length (*P* = 0.68) separately to the model, did not show significant associations between them and the prevalence of DED (definition #4).

**Table 5:** Associations (multivariable analysis) between the prevalence of dry eye disorder definition #2: dry eye symptoms score was ≥ 2 and Schirmer´s test of < 5 mm or the prevalence of dry eye disorder (definition #4; dry eye symptoms score was ≥ 1, Schirmer test ≤ 5 mm, and a Meibomian gland dysfunction grade of 1 (telangiectasia at the lid margin) or higher) and ocular and systemic parameters in the Ufa Eye and Medical Study.

Parameter	Measurement unit	Definition #2		Definition #4	
		Standardized regression coefficient or odds ratios (95% confidence intervals)	*P*-value	Standardized regression coefficient or odds ratios (95% confidence intervals)	*P*-value
Age	Years	0.98 (0.97, 1.00)	0.005		
Sex	Men/women	2.04 (1.61, 2.58)	< 0.01	1.71 (1.31, 2.22)	< 0.001
Region of habitation	Rural/urban	1.24 (1.01, 1.54)	0.045		
Depression score		1.04 (1.01, 1.07)	0.006	1.04 (1.01, 1.07)	0.009
Number of days per week with fruit intake	0–7	1.07 (1.02, 1.13)	0.008	1.06 (1.00, 1.13)	0.048
History of thyroid disease	Yes/no	1.49 (1.12, 1.99)	0.006	1.63 (1.19, 2.24)	0.002
History of angina pectoris	Yes/no	1.50 (1.11, 2.02)	0.008		
Serum concentration of urea	mmol/L	1.12 (1.06, 1.19)	< 0.001	1.18 (1.11, 1.25)	< 0.001
Serum concentration of bilirubin	mmol/L			0.98 (0.97, 1.00)	0.01
Monocyte percentage	Percentage if leukocyte count			1.09 (1.04, 1.14)	< 0.001
Intraocular pressure	mm Hg			0.97, 0.94, 1.00	0.03

The prevalence of DED (definition #2) was associated with younger age, female sex, urban region of habitation, higher depression score, higher number of days per week with fruit intake, higher prevalence of a history of thyroid disease, angina pectoris and thyroid disease, and higher serum concentration of urea (Table 5) Adding the parameter of body mass index (*P* = 0.18), waist-hip circumference ratio (*P* = 0.08), physical activity score (*P* = 0.&§), prevalence of current smoking (*P* = 0.&(), level of education (*P* = 0.86), prevalence of diabetes mellitus (*P* = 0.87), serum concentration of bilirubin (*P* = 0.33), monocyte percentage (*P* = 0.08), intraocular pressure (*P* = 0.17), and axial length (*P* = 0.08) separately to the model, did not show significant associations between them and the prevalence of DED (definition #2).

## Discussion

In our population-based study from Russia, a Schirmer´s test of ≤ 5 mm in the worse eye was found in 21.3% of the study participants, and the mean score of DED was 1.37 ± 1.82. The prevalence of MGD of any degree was 52.6%. A higher DED prevalence (definition #2) correlated with parameters such as younger age, female sex, urban region of habitation, higher depression score, and higher prevalence of a history of thyroid disease, while determinants of a higher prevalence of DED (definition #4) were female sex, higher depression score, higher prevalence of a history of thyroid disease, higher serum concentration of urea and lower bilirubin concentration, and lower intraocular pressure (Table 5).

The findings obtained in our study population can be compared with observations made in other study samples. In the Iranian Shahroud Eye Cohort Study, the prevalence of MGD, defined as recommended by the International Workshop on MGD, was 26.3%^14^. A higher MGD prevalence was associated with a higher prevalence of pinguecula and arterial hypertension, lower prevalence of diabetes mellitus, lower serum concentrations of high-density lipoprotein and less years of education^14^. In a hospital-based study on Japanese patients aged 50 + years and scheduled for cataract surgery, the prevalence rates of symptomatic MGD and total (symptomatic + asymptomatic) MGD were 18.0% and 47.5%, respectively^15^. The MGD prevalence increased with older age. In a comprehensive meta-analysis of globally available population-based studies on the DED prevalence, the latter ranged between 5 and 50%^9^. A meta-analysis of published studies on the MGD prevalence revealed a figure of 36% (95% CI 24, 50), with a higher rate in men than in women^12^. Akowuah and colleagues performed a meta-analysis of hospital-based studies and reported a MGD prevalence of 45.9% for Africa^34^. In the Singapore Malay Eye Study, the MGD prevalence was 56.3% (Table 6)^35–43^.

**Table 6:** Previous studies on the prevalence of dry eye disease and Meibomian gland dysfunction.

Study		Number of participants	Methods	Prevalence	Associations
Vehof et al. ^36^	Population-based Lifelines Cohort Study in the Netherlands	79,866	Women's Health Study dry eye questionnaire	9.1%; Prevalence of dry eye symptoms were particularly prevalent in 20–30 years olds;	Dry eye correlated associated with comorbidities in almost all body systems and with various risk factors, such as female sex, contact lens use, irritable bowel syndrome, fibromyalgia, chronic fatigue syndrome, eye surgery including cataract and laser refractive surgery, keratoconus, osteoarthritis, connective tissue diseases, atherosclerosis, Graves' disease, autistic disorder, depression, 'burnout', Crohn's disease, sarcoid, lichen planus, rosacea, liver cirrhosis, sleep apnea, sinusitis, thyroid function, and air pollution (NO2)
Farrand et al.^37^	Cross-sectional, population-based survey	75,000 participants in the 2013 National Health and Wellness Survey		6.8% of the US adult population was projected to have diagnosed DED;	DED prevalence increased with age and female sex, but not with ethnic background, level of education, or region
Yang et al. ^38^	Cross-sectional survey	2140	Ocular Surface Disease Index (OSDI) and the Women's Health Study (WHS) questionnaire	34.4% with an OSDI score > 22; 23.5% had dry eye according to the WHS	Female sex, contact lens wear, screen use for more than 6 h per day, less than 6 h of sleep a night, and various medications
Akowuah et al.^39^	Literature search			Overall prevalence of DED in Africa: 42.0%)	No associations with sex, type of study, country, study population and the diagnostic criteria used
Alkabbani et al.^42^	Online survey to university associates	452	Ocular Surface Disease Index	DED prevalence: 62.6% (283/452)	
Sherry et al. ^40^	Population-based cross-sectional study	602	Ocular Surface Disease Index (OSDI) questionnaire	DED: 36.4% (OSDI score ≥ 13 (mild to moderate and severe OSDI status))	
Stapleton et al.^9^	Meta-analysis of literature;			DED prevalence: 5 to 50%	Older age, female sex, Asian ethnicity
Shanti et al. ^41^	Cross-sectional	769	Interviewer-assisted Ocular Surface Disease Index (OSDI) questionnaire; tear film break-up time, fluorescein corneal staining and Schirmer test	DED prevalence: 64% (95% CI 60.6–67.3)	Female gender and older age
Hassanzadeh et al.^13^	Meta-analysis			MGD prevalence: 0.358 (95% CI 0.26–0.46); MGD prevalence in clinical studies: 0.358 (95% CI 0.24–0.50) an d in population-based studies: 0.359 (95% CI 0.22–0.52)	Male sex; higher in Arabs (71.0%) and Hispanics (67.5%) than in Africans (21.2%) and Caucasians (29.5%)
Akowuah et al.^34^	Meta-analysis of hospital-based studies	4963		Overall prevalence of MGD in Africa: 45.9% (95% CI 27.6–64.1%)	No association of MGD prevalence with sex and study setting
Amano et al. ^15^	Hospital-based	510 consecutive patients scheduled for cataract aged 50 + years	Symptoms-related questionnaire and comprehensive slit-lamp examination	Prevalence of symptomatic MGD and total (symptomatic + asymptomatic) MGD: 18.0% and 47.5%, respectivel	Prevalence of total MGD: older age
Siak et al.^35^	Population-based	Urban Malay population in Singapore	Slit lamp-based clinical examination	Age-standardized MGD prevalence: 56.3% (95% CI 53.3–59.4)	Male sex, higher prevalence of pinguecula, higher diastolic blood pressure, and use of angiotensin II receptor blockers

The DED prevalence ranged in previous studies between 9 to 30%, when symptoms combined with signs were taken into account, and it ranged between 7 to 52%, when only symptoms were considered^2,9–11^. In the population-based Lifelines Cohort Study in the Netherlands on almost 80,000 individuals, the DED prevalence was 9.1%, with a higher figure in persons aged 20–30 years^36^. In the cross-sectional population-based 2013 National Health and Wellness Survey performed in the US on 75,000 participants, 6.8% of the adult population was diagnosed with DED^38^. In the study performed by Yang and associates in 2140 participants, 34.4% had an ocular surface disease index (OSDI) score of > 22, and 23.5% had DED according to the Women's Health Study questionnaire^38^. The results of our study can be compared also with the findings made in the Ural Very Old Study which was conducted in similar study regions on a population aged 85 + years^33^. In that investigation, the prevalence of a Schirmer´s test of ≤ 5 mm was 34.3%, and the mean prevalence of MGD grade 1, 2, 3, 4 and of any grade was 31.4%, 26.4%, 7.6%, 3.3% and 68.8%, respectively. The prevalence of DED using the diagnosis definition #1 to #5 was 18.2%, 14.5%, 8.1%, 14.8%, and 7.4%, respectively. A higher DED prevalence (definition #2) was associated with female sex, rural region of habitation, longer axial length, higher hearing loss score and lower self-reported salt consumption^33^. A higher prevalence of DED in association with a MGD (definition #4) was correlated with rural region of habitation, lower salt consumption and higher hearing loss score. The prevalence of MGD of any grade was 52.6% (95% CI 51.1, 54.1) in our study population. That figure was lower than the MGD prevalence in the Ural Very Old Study (MGD prevalence of any grade: 68.8%), and it was higher than the rates reported in previous studies, in which the MGD prevalence, pooled in a meta-analysis, was 36% and ranged between 5 and 50%^9,12–15,33^.

In our study population, a higher DED prevalence of both DED definitions #2 and #4 was associated with female sex. The prevalence of DED based on definition 2 increased slightly with younger age, while the prevalence of DED based on definition #4 was not related with age. In previous investigations, the DED prevalence increased with older age^9^. In the meta-analysis performed by Stapleton and colleagues in 2017, the DED prevalence also was higher in women than in men, with this inter-sex difference increasing with older age^9^. In previous investigations, Asian ethnicity was associated with a higher DED prevalence. In our population, however, Russian ethnicity versus non-Russian (i.e., Central Asian) ethnicity was not related with the prevalence of DED and MGD. The reason for the discrepancy between the studies might have been that the Asian population group in our study population was composed of Central Asians, such as Bashkirs and Tartars, in contrast to East Asians, who were included in most of the previous studies on Asian populations and who often were living in megacities. Otherwise, it has remained unclear why in our study population the prevalence of DED as based on definition 2 slightly increased with younger age, while in contrast the prevalence of DED based on definition #4 was not related with age.

The finding of an association between a higher DED prevalence and a higher depression score in our study population agrees with observations made in previous epidemiologic studies^32,33,44–47^. In a national veterans population study, Galor and colleagues examined the association between DED and psychiatric disorders and found that depression was associated with a twofold increased risk of having DED^32^. Kim and associates also reported in their population-based cross-sectional study a positive correlation between depression and dry eye symptoms^46^. In the Dry Eye Assessment and Management (DREAM) study, a recent multicenter randomized clinical trial of 535 patients with moderate to severe DED, individuals with depression had worse DED symptoms, and a higher degree of depression was associated with worse DED symptoms^48^. The DREAM study had primarily been designed to evaluate the effect of omega-3 supplements on the symptoms and signs of dry eye disease relative to placebo supplement^49^. In the same investigation, more severe DED signs were associated with a higher prevalence of Sjögren syndrome (*P* < 0.001), facial rosacea (*P* = 0.002), rheumatoid arthritis (*P* = 0.002), peripheral artery disease (*P* < 0.001), and daily smoking history (*P* = 0.047)^50^. In other smaller scaled case control investigations, the scores of depression and anxiety were associated with DED signs^51^. In contrast to our study, DED did not correlate with the prevalence of thyroid dysfunction, while, as in our study, it was neither associated with the prevalence of osteoarthritis, diabetes, hypercholesterolemia, hypertension, and hypertriglyceridemia.

While the present study as also previous investigations showed associations between a higher prevalence of DED with disorders such as depression, sex and thyroid disease, our study can, due to its cross-sectional character and design, not explore the causes for such relationships. In the case of depression, the subjective threshold of experiencing a given ocular surface state as dry may be altered as may the blinking frequency be affected. Similar reasons may hold true in the case of thyroid disease and its association with DED. Future longitudinal studies may explore the causality of these relationships.

When the results of our study are discussed, its limitations should be taken into account. First, we did not assess the break-up time of the tear film, a parameter that has been included in previous studies on the prevalence of DED. Second, 5899 (80.5%) out of 7328 eligible individuals participated in the Ural Eye and Medical Study, and out of these 5899 individuals, 5153 (87.4%) persons underwent the assessments of DED and MGD. The missing data of the individuals not participating in the study might have led to a bias. Third, the study regions of the Ural Eye and Medical Study were characteristic for Southern Russia in terms of demography, geography and climate. In terms of the ethnic background, the percentage of Russians was lower in our study population than in North-Western Russia and Central Russia. In the multivariable analysis, however, the ethnic background was not associated with the prevalence of DED and MGD, so that the relatively high percentage of non-Russians on the total study population may not have markedly influenced the results. Fourth, various definitions of DED and MGD have been used in previous investigations^52–55^. To address that problem, we used four different definitions of DED to make the results of our study comparable with those of previous investigations. Fifth, the study population included only individuals aged 40 + years, so that the study results cannot be transferred on the younger population. Strengths of our investigation are that it is the first population-based investigation on the prevalence of DED and MGD in Russia and Eastern Europe, and that a multitude of systemic parameters was assessed and included in the statistical multivariable analysis.

In conclusion, a higher DED prevalence (mean: 8.3%) and MGD prevalence (any grade: 52.6%) in this population from urban and rural Russia was associated with female sex, thyroid disease, and higher depression score.

## Data Availability

All identified data are available upon reasonable request from the corresponding author.
